# Effect of education and clinical assessment on the accuracy of post partum blood loss estimation

**DOI:** 10.1186/1471-2393-14-110

**Published:** 2014-03-19

**Authors:** Hanan M Al-Kadri, Hanan Dahlawi, Mona Al Airan, Elham Elsherif, Nasser Tawfeeq, Yane Mokhele, Drika Brown, Hani M Tamim

**Affiliations:** 1 Department of Obstetrics and Gynaecology, King Abdulaziz Medical City, King Saud bin Abdulaziz University for Health Sciences, PO Box 57374, Riyadh 11574, Saudi Arabia; 2 Department of Anaesthesia, King Abdulaziz Medical City, King Saud bin Abdulaziz University for Health Sciences, Riyadh, Saudi Arabia; 3 Nursing Services, King Abdulaziz Medical City, Riyadh, Saudi Arabia; 4 Department of Medical Education, College of Medicine, King Saud bin Abdulaziz University for Health Sciences, Riyadh, Saudi Arabia

**Keywords:** Post partum blood loss, Visual estimation, Maternal mortality, Maternal morbidity, Education

## Abstract

**Background:**

This research aimed to assess the effect of health care provider education on the accuracy of post partum blood loss estimation.

**Methods:**

A non-randomized observational study that was conducted at King Abdulaziz Medical City, Riyadh, Saudi Arabia between January 1, 2011 and June 30, 2011. Hundred and twenty three health care providers who are involved in the estimation of post partum blood loss were eligible to participate. The participants were subjected to three research phases and an educational intervention. They have assessed a total of 30 different simulated blood loss stations, with 10 stations in each of the research phases. These phases took place before and after educational sessions on how to visually estimate blood loss and how to best utilize patient data in clinical scenarios. We have assessed the differences between the estimated blood loss and the actual measure. *P*-values were calculated to assess the differences between the three research phases estimations.

**Results:**

The participants significantly under-estimated post partum blood loss. The accuracy was improved after training (*p*-value < 0.0001) and after analysing each patient’s clinical information (*p*-value = 0.042). The overall results were not affected by the participants’ clinical backgrounds or their years of experience. Under-estimation was more prominent in cases where more than average-excessive blood losses were simulated while over-estimations or accurate estimations were more prominent in less than average blood loss incidents.

**Conclusion:**

Simple education programmes can improve traditional findings related to under-estimation of blood loss. More sophisticated clinical education programmes may provide additional improvements.

## Background

Guidelines on the management of postpartum haemorrhage (PPH) have reiterated the importance of the accurate estimation of blood loss [1,2]. Several methods of measuring blood loss have been proposed. In general, these methods should be accurate, practical, consistent, readily communicated and easily incorporated into most labour ward protocols [3-5].

However, questions remain about how to best guarantee the accurate estimation of blood loss by health care providers. Previous studies have proposed several blood loss assessment methods that aim to improve the accuracy of blood loss estimation [3,6,7]. So far, none of these methods alone have proved adequate to guarantee patient safety.

Despite being notoriously inaccurate [8], the visual estimation of blood loss is widely used because of its relative ease [9]. The degree of its inaccuracy varies between 30 to 50% underestimation of actual loss [3,4,10]. Importantly, this inaccuracy increases with increasing blood loss in situations where maternal lives are at risk [3]. When translated into clinical practice, such underestimations of blood loss may delay or deter identification and diagnosis of major PPH, resulting in catastrophic outcomes [11].

We have conducted this study to answer three questions: (1) To what extent are health care providers in a tertiary care centre accurate in their visual estimation of blood loss? (2) Will education on how to estimate blood loss improve their estimation accuracy? (3) Will further education on how to utilise a patient’s clinical information further improve this accuracy?

## Methods

A non-randomized observational study was conducted at King Abdul Aziz Medical City, Riyadh (KAMC-R), Saudi Arabia between January 1, and June 30, 2011. All health care providers who are involved in the estimation of post partum blood loss including obstetricians, anaesthetists, nurses and midwives were eligible and were invited to participate through e-mail and direct invitations. Approximately 300 health care providers were eligible to participate in this research. Those who agreed to participate have signed a written informed consent. The participants were asked to give information regarding their job title and length of experience. These variables were used to compare between the different participants groups (nurses versus physicians & less than ten years versus more than 10 years of clinical experience). Ethical approval was obtained from the King Abdullah Medical International Research Centre ethics committee.

### Research phases and intervention

**Figure 1:**
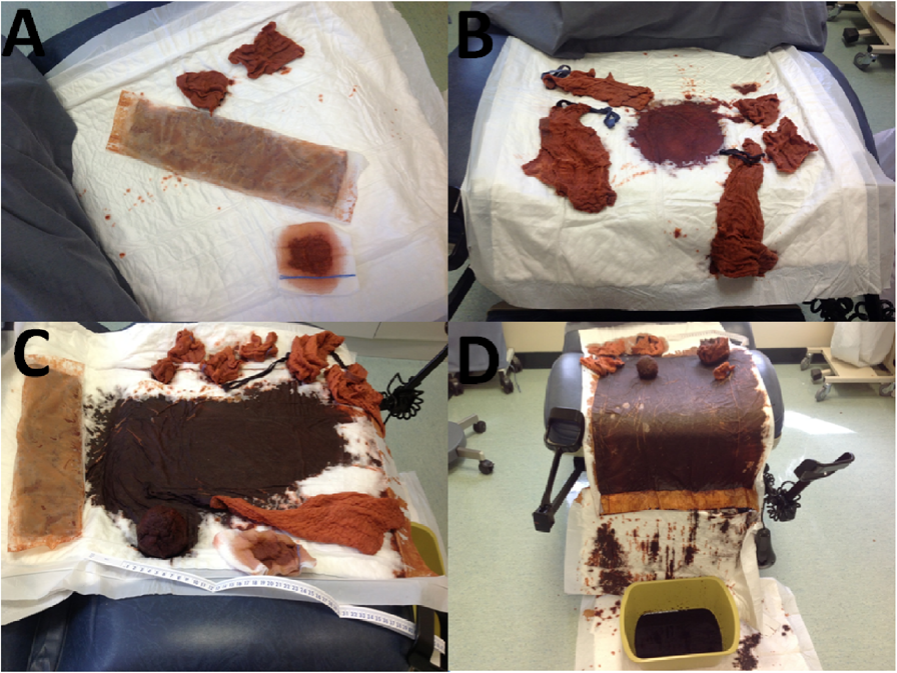
Simulated blood loss stations representing: A, less than average blood loss (250 ml); B, average blood loss (500 ml); C, more than average blood loss (950 ml) and D, excessive blood loss (2000 ml).

This study included three different phases and one primary intervention. During each phase, ten blood loss simulation stations were designed aiming to assess predetermined simulated blood loss. Grape jelly, starch, glucose, sugar, artificial colours, and other materials were used to simulate clots and blood. We have avoided the use of true blood for hygienic and infection control reasons. The simulated blood loss stations were presented in a clinical setup and were ranged between 200 and 2000 ml to simulate less than average, average, more than average, and excessive blood losses [8,12]. Simulated blood quantities; liquid and clot like, were placed on sanitary pads, delivery pads, basins, drapes, sheets and on the floor (Figure 1).

#### Phase one

This phase aimed to assess a participant’s baseline accuracy of visual blood loss estimation. The participants were asked to pass individually from one simulation station to the other in a timely, standardised fashion. Each of the ten simulated blood loss stations was placed in a clinic room and participants were allowed a minute to estimate blood loss of each station. They have documented their visual estimation of the blood loss of each station on a special form and placed it in a special box after finalising the tenth station.

#### Research intervention: health care professional education programme

After all the participants completed phase one, we implemented the research intervention. We have conducted education sessions on how to accurately assess blood loss over a period of two months. We have accommodated the participants schedule, work commitments and other circumstances. Occasionally we have educated the participants in small groups of two to three until we guaranteed that all the included health care providers have successfully attended the planned education intervention. Power Point presentation with pictures illustrations as well as simulated blood losses on large gauze, 4X4 gauze. sheets, chux and others were used. Through the used power point presentation and blood loss simulations the participants were taught about the importance of accurate estimation of blood loss, the patho-physiology of PPH, various known methods to estimate post partum blood loss, visual blood loss estimation methods with clear pictures and examples. The presentation have included also education on the utilisation of patients’ clinical condition data to enhance the accuracy of blood loss estimation. Clinical indicators such as patients pulse, blood pressure, haemoglobin concentration prior to bleeding, level of alertness and others were presented and these indicators significance in calculating blood loss and initiating actions were clarified. Practical training sessions using simulated blood have followed each presentation. The participants were given time to ask questions, clarify any vague idea and practice on examples.

#### Phase two

This phase aimed to assess the effects of education on the accuracy of simulated blood estimation. We conducted this phase after we confirmed that all participants have attended the planned teachings of the research intervention. During this phase, we performed second set of sessions of ten simulated blood loss stations. Each one of the participants have estimated the blood loss of phase two stations. We have maintained the same amount and design of simulated blood loss for each station as they were in phase I of the research.

#### Phase three

This phase aimed at assessing the effect of both education and the utilisation of patients’ clinical information on the accuracy of blood loss estimation. Utilising the same method described above, the same group of participants have estimated for a third time the simulated blood loss using a clinical scenario prepared to suit the planned different blood loss stations. In each scenario, a clinical case summary similar to the one usually presented by the attending health care provider to his/her senior when they call for help is given. These scenarios included the patients’ parity, gestational age, haemoglobin concentration on admission, method of delivery, complications and the patients’ clinical assessment findings. The participants were advised to read the scenario assigned for each blood loss station and to utilise the given information while visually estimating the blood loss.

Each one of the 3 phases of this research was conducted several times to accommodate the participants work commitments and to make sure that all of them have attended all of the phases. The methodology of stations design and the amount of simulated blood loss in each station were identical during all study phases.

To avoid any effect of the repeated estimation attempts on the participants performance, the accurate estimation of blood in the ten stations was kept blinded for all the participants during all the research phases. Furthermore, the participants were randomly assigned to the research stations and have performed the different research phases with different stations order.

#### Statistical analysis

Data was entered into the Statistical Package for Social Sciences software version 16. We calculated the number and percentage for the categorical variables and the mean and standard deviation (sd) for the continuous variables. Univariate analyses for the association between real simulated blood loss and the participants’ visual estimation of simulated blood loss at the three different research phases were performed. We calculated the difference between the estimated blood loss and the actual measure. A negative value for this difference would indicate under-estimation, whereas a positive one would indicate an over-estimation. *P*-values were calculated using the chi-squared test or Student’s *t*-test, as appropriate.

## Results

A total of 123 eligible participants who had various levels of experience and various clinical backgrounds completed this study. The baseline characteristics of the study participants are summarised in Table 1. The average age of participants was 39.3 years (sd = 10.0), with the majority (93.5%) being females. This participants gender finding is comparable to the total number of female represented within the eligible population (95.5%). We have identified also that 76.2% of the participants were nurses, while physicians represented 23.8% of the study population. This participants profession finding is comparable to the 74% total number of nurses within the eligible population. Finally, there was an equal distribution among participants in terms of their years of experience, being < or ≥ 10 years (47.7% and 52.3%, respectively).

**Table 1:** Baseline characteristics of study participants

		**Mean (sd)**
Age		39.3 (10.0)
		Number (%)
Gender	Male	8/123 (6.5%)
	Female	115/123 (93.5%)
Nationality	Saudi	24/123 (19.5%)
	Non-Saudi	99/123 (80.5%)
Title	Physician	29/123 (23.6%)
	Nurse	94/123 (76.4%)
Experience	<10 years	59/123 (48.0%)
	≥10 years	64/123 (52.0%)

Sd = Standard deviation.

**Figure 2:**
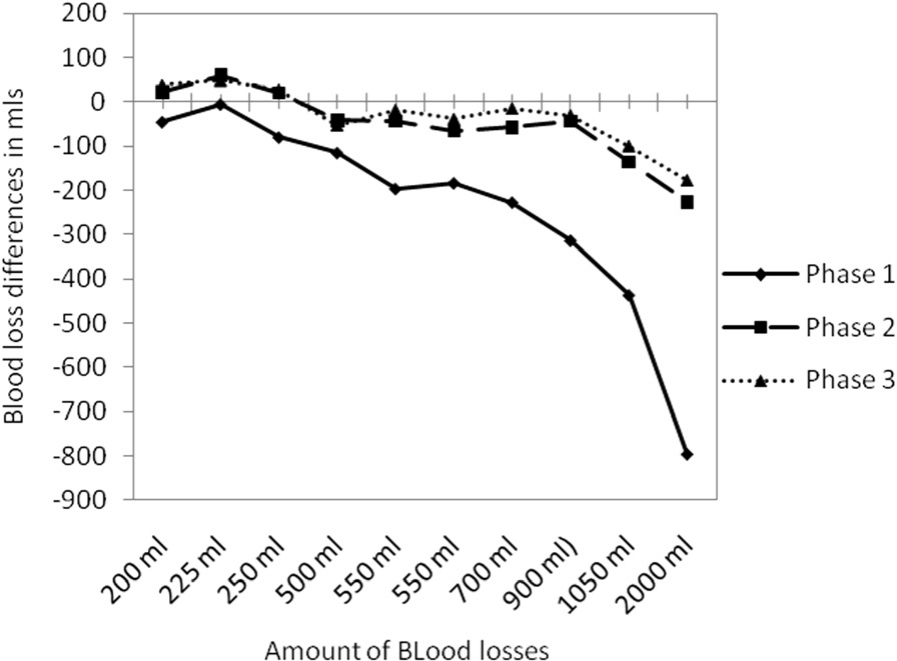
Comparison between the mean differences of blood loss estimation for the 10 different stations over the three research phases arranged in an ascending manner.

Figure 2 represents a comparison between the mean differences between the actual blood losses and estimated blood losses of the ten simulated blood loss stations over the three research phases. In this figure we can identify clearly the significant improvement of blood loss estimation between Phase 1 and Phase 2, while the difference is minimal between phase 2 and phase 3.

Table 2 represents the level of accuracy of simulated blood loss estimations for the three research phases. It shows the mean differences between the actual blood losses and the estimated blood losses in the different research stations and throughout the different research phases. The statistically significant differences between phases 1 and 2, and phases 1 and 3 mean differences were identified. We found that the participants tend to under-estimate simulated blood loss for all 10 stations at the baseline (phase 1). The least accurate estimates were found to take place at stations 5 and 10 (-795.7 and -436.5, respectively), which represent the stations with the highest actual blood loss simulations (2000 ml and 1050 ml, respectively). On the other hand, the most accurate measurements took place at station 3 (-5.2) which represented the station with the lowest actual simulated blood loss (225 ml).

**Table 2:** Degree of accuracy of blood loss estimation for the 3 phases, with p-values for the association between phase 1 and phase 2, and phase 2 and phase

	**Phase**			**P value**	
	**P1 MD (sd)**	**P2 MD (sd)**	**P3 MD (sd)**	**P1 vs P2**	**P2 vs P3**
Station 1 (200 ml)	-45.2 (93.0)	21.7 (89.5)	37.7 (119.2)	<0.0001	0.16
Station 2 (225 ml)	-5.2 (135.1)	59.6 (115.9)	49.2 (102.3)	<0.0001	0.18
Station 3 (250 ml)	-80.0 (112.0)	19.9 (97.5)	27.6 (122.0)	<0.0001	0.40
Station 4 (500 ml)	-115.0 (291.0)	-42.1 (309.4)	-52.4 (202.1)	0.05	0.70
Station 5 (550 ml)	-196.6 (241.7)	-44.1 (182.2)	-18.0 (185.1)	<0.0001	0.11
Station 6 (550 ml)	-183.7 (254.4)	-65.7 (237.3)	-38.6 (205.5)	<0.0001	0.13
Station 7 (700 ml)	-227.4 (259.3)	-57.9 (235.1)	-14.2 (205.9)	<0.0001	0.02
Station 8 (900 ml)	-313.3 (317.5)	-42.9 (398.0)	-29.9 (360.3)	<0.0001	0.66
Station 9 (1050 ml)	-436.5 (505.4)	-135.8 (352.3)	-100.1 (342.6)	<0.0001	0.18
Station 10 (2000 ml)	-795.7 (729.6)	-227.1 (823.4)	-178.3 (634.2)	<0.0001	0.36

P = Phase.

P-value significant ≤ 0.05.

MD = Mean Difference.

sd = Standard deviation.

Stations were ordered in an ascending order of simulated blood loss volumes, although the sequence was random for the different phases conduction.

The accuracy of simulated blood loss estimation in phases 2 and 3 was found to be better for all of the stations as the differences between the estimated and actual values had decreased. In both phases 2 and 3, there was an under-estimation for stations 1, 4, 5, 6, 7, 8, and 10 where average, more than average or excessive blood losses were simulated. Likewise, there was an over-estimation for stations 2, 3, and 9; which were the stations where the lowest actual levels were simulated. An examination of the comparisons between the different research phases revealed that the difference between phases 1 and 2 was statistically significant for all 10 stations (*p*-value ≤ 0.05). On the other hand, the comparison between phases 2 and 3 revealed no significant difference for any station except for station 1 (*p*-value = 0.02).

Considering the overall results of the research, it was found that the overall differences for phase 1, 2 and 3 (differences between the mean actual loss and the mean estimated loss) were -2398.6 (sd = 2146.1), -514.2 (sd = 1898.4), and -317.1 (sd = 1666.2) ml, respectively. Statistically significant differences were found for the differences between phase 1 and 2 mean simulated blood loss (*p*-value < 0.0001), phase 1 and 3 (*p*-value < 0.0001) and phase 2 and phase 3 (*p*-value = 0.042). These results indicate that there was a significant effect of education on reducing errors of blood loss estimations. Moreover, there was a significant, though less prominent, effect of education and the utilisation of patients’ clinical data on the accuracy of simulated blood loss estimation.

The effect of experience on the accuracy of blood loss estimation was assessed in Table 3. There was no consistent better or worse trend on the participants’ estimation within the three different research phases. This was unfailing with the exception of phase 1, station 5 where an excessive blood loss of 2000 ml was simulated and estimated more accurately by the more experienced health care providers (*p*-value = 0.01). Moreover, a positional effect, whether being a physician or a nurse, on the accuracy of visual estimation of simulated blood loss was assessed and found to be insignificant (Table 4).

**Table 3:** Effect of years of experience on the degree of accuracy of blood loss estimation for the research three phases

	**Phase 1**			**Phase 2**			**Phase 3**		
	**<10 yrs MD (sd)**	**≥10 yrs MD (sd)**	**P-value**	**<10 yrs MD (sd)**	**≥10 yrs MD (sd)**	**P-value**	**<10 yrs MD (sd)**	**≥10 yrs MD (sd)**	**P-value**
Station 1 (200 ml)	-55.1 (81.8)	-51.8 (96.8)	0.86	38.8 (102.2)	16.0 (95.9)	0.29	51.5 (92.0)	43.9 (168.1)	0.80
Station 2 (225 ml)	-32.0 (133.3)	18.6 (148.8)	0.10	68.2 (124.1)	62.7 (134.4)	0.85	59.0 (109.5)	42.8 (101.2)	0.48
Station 3 (250 ml)	-87.0 (85.3)	-70.6 (145.3)	0.53	15.7 (71.3)	24.0 (113.6)	0.69	28.7 (94.0)	22.7 (127.2)	0.81
Station 4 (500 ml)	-151.8 (255.0)	-78.6 (374.5)	0.30	-57.6 (215.2)	-39.7 (367.2)	0.79	-38.4 (216.9)	-74.6 (213.7)	0.44
Station 5 (550 ml)	-210.0 (169.7)	-209.2 (302.4)	0.99	-19.3 (147.0)	-69.8 (176.1)	0.16	-31.2 (131.8)	-46.0 (223.0)	0.71
Station 6 (550 ml)	-195.9 (194.4)	-151.2 (338.5)	0.46	-86.6 (253.4)	-73.9 (250.5)	0.82	-15.0 (234.6)	-89.1 (192.5)	0.11
Station7 (700 ml)	-267.8 (219.9)	-226.1 (265.7)	0.43	8.8 (267.9)	-105.8 (242.3)	0.04	25.0 (234.3)	-60.5 (209.1)	0.08
Station 8 (900 ml)	-351.8 (283.0)	-283.5 (352.4)	0.33	22.2 (373.2)	-85.7 (414.9)	0.21	6.8 (319.5)	-95.6 (354.9)	0.17
Station 9 (1050 ml)	-475.7 (318.3)	-347.3 (734.9)	0.30	-132.6 (304.6)	-236.1 (398.0)	0.18	-48.0 (343.1)	-184.4 (341.4)	0.07
Station 10 (2000 ml)	-1032.1 (527.2)	-652.2 (804.0)	0.01	-290.2 (708.3)	-271.5 (890.2)	0.92	-188.6 (682.8)	-230.9 (690.2)	0.78

Yrs = Years.

P-value significant ≤ 0.05.

MD = Mean Difference.

sd = Standard deviation.

Stations were ordered in an ascending order of simulated blood loss volumes, although the sequence was random for the different phases conduction.

**Table 4:** Effect of position (physician vs. nurse) on the degree of accuracy of blood loss estimation for the research three phases

	**Phase 1**			**Phase 2**			**Phase 3**		
	**Physician MD (sd)**	**Nurse MD (sd)**	**P-value**	**Physician MD (sd)**	**Nurse MD (sd)**	**P-value**	**Physician MD (sd)**	**Nurse MD (sd)**	**P-value**
Station 1 (200 ml)	-35.2 (96.1)	-49.8 (91.5)	0.46	40.0 (119.4)	16.5 (78.3)	0.22	28.3 (84.0)	40.5 (129.1)	0.63
Station 2 (225 ml)	-4.7 (141.8)	-8.4 (131.3)	0.89	94.3 (143.8)	48.9 (105.0)	0.07	103.4 (149.2)	33.1 (76.5)	0.001
Station 3 (250 ml)	-64.8 (141.6)	-85.0 (102.2)	0.40	48.4 (117.6)	11.7 (89.6)	0.08	84.5 (158.3)	10.4 (103.8)	0.004
Station 4 (500 ml)	75.2 (447.7)	-172.4 (190.2)	<0.0001	20.9 (194.6)	-61.1 (337.3)	0.22	39.1 (195.1)	-80.5 (197.8)	0.005
Station 5 (550 ml)	-215.3 (175.7)	-197.7 (251.8)	0.73	7.8 (198.1)	-59.4 (175.8 )	0.08	61.4 (229.9)	-42.1 (163.5)	0.008
Station 6 (550 ml)	-157.6 (247.2)	-192.3 (258.7)	0.53	-44.0 (261.3)	-70.7 (231.1)	0.60	-5.2 (193.1)	-48.1 (210.1)	0.33
Station7 (700 ml)	-238.6 (240.1)	-234.9 (245.0)	0.94	-56.2 (215.9)	-57.9 (243.0)	0.97	-57.4 (211.8)	-1.9 (204.2)	0.21
Station 8 (900 ml)	-243.9 (366.4)	-337.3 (300.4)	0.17	119.0 (445.1)	-89.8 (371.6)	0.01	96.7 (440.9)	-67.6 (325.9)	0.03
Station 9 (1050 ml)	-444.1 (340.2)	-434.0 (550.9)	0.93	-15.7 (350.5)	-169.8 (347.0)	0.04	-65.5 (265.2)	-112.5 (365.3)	0.52
Station 10 (2000 ml)	-648.3 (785.0)	-871.7 (648.6)	0.13	-8.6 (649.7)	-290.1 (865.5)	0.11	45.8 (616.1)	-246.9 (630.2)	0.03

MD = Mean Difference.

sd = Standard deviation.

Stations were ordered in an ascending order of simulated blood loss volumes, although the sequence was random for the different phases conduction.

## Discussion

The results of this research indicate that health care providers in a tertiary health care centre were inaccurate and significantly under-estimating blood loss volumes when visually assessing simulated blood loss. Such estimations were improved after training sessions. Borderline statistically significant differences were found after utilising the patients’ clinical information to judge the amount of blood lost (*p*-value = 0.042). The overall results were not affected by the participants’ length of experience except when the simulated blood loss was excessive; in such cases, health care providers with longer experience duration were more accurate in their estimations. This difference disappeared in phases 2 and 3 after educational measures. The overall results were also not affected by the participants’ clinical backgrounds of being either nurses or physicians.

The blood loss simulation stations that were used to train and assess participants in this research have proved their effectiveness; these stations helped to improve the knowledge and skills of health care providers on how to best assess post partum blood loss accurately (Table 2). Although the simulations were successfully utilised, other traditional training methods have proved equally effective in the acquisition of similar knowledge and skills in various obstetric emergencies. For example, studies of case scenarios with high resolution pictures or computer slides representing real or simulated blood loss can be used to replace the simulated blood loss stations, reducing the need for consumables and expensive teaching or assessment methods [13].

The impact of education on the accurate estimation of post partum blood loss has been stressed in many official recommendations [1,2] and international publications [12-19]. It was identified as a major contributor to the improvement of maternal safety and the reduction of PPH-related morbidities. Despite these strong and repeated recommendations, the strategy and content of this education was not clear whether it should include only training on the visual estimation of blood loss, other methods of estimation, or it should include strategies to utilise patient clinical assessment data [20]. In general, all of the existing classifications of PPH, such as the WHO and Benedetti’s, utilise different criteria, but share the requirement for correct and timely estimations of blood loss [20,21].

Quantitative methods to estimate volume of blood loss included direct collection of blood into containers, the gravimetric assessment of blood loss [6], measuring blood loss by 51Cr-tagged erythrocytes [22], calculation of changes in blood indices before and after delivery, acid hematin method [23] and plasma volume calculation before and after delivery using radioactive tracer. These methods are difficult to be adopted in clinical practice due to their complicated nature, time consuming, delayed results, expenses and lack of practicality. On the other hand visual estimation of blood loss is found to be easy, fast, approachable, and cheap method as compared to all other quantitative methods of blood estimation [3-5]. Having education appears to carry significant effect on improving blood loss estimation accuracy and reliability [6,19,24], it seems that visual estimation of blood loss can be the method of choice to be used in labour and delivery setups if appropriate training and education are implemented.

It is evident from this research that health care providers dealing with delivering mothers should be trained to accurately estimate blood loss. In fact, attending similar training programme should be recommended as a pre-requisite to caring for women in labour. The benefits of visual blood loss estimation education programme are expected to outweigh its financial costs and time commitments. These programmes are expected to significantly reduce post partum blood loss errors and result in early alertness, and timely actions on cases of bleeding. These programmes may also result in reduction of both maternal morbidities and mortalities [17,19,24]. In fact this is an area that requires future research to be confirmed. Regardless the education strategy used, absence of real patient management stress, time pressure and the use of artificial blood soaking known gauze and sheets size with measures or scales assistance may have some effect on estimation accuracy promotion [6]. Real life or in vivo stressful scenarios might not be as simple as the in vitro ones utilized despite the effort to simulate true PPH field.

More advanced levels of training that utilise patients’ clinical conditions in alerting health care providers and planning further actions on bleeding added extra benefits to the primary education in our research. Although this addition may enhance the accuracy of PPH estimation and its timely management [8] but its time consuming nature, the very limited improvement in blood loss accuracy assessment and its requirement to more advanced knowledge and training could limit its use in low resources setups. Education on utilising patients’ clinical conditions is may be advised for a tertiary care centres where more support, resources and trained staff are available.

### Study limitations

Although we recruited approximately 40% of the involved stakeholders in this study, it is possible that those who were involved are either better or worse in their abilities to estimate blood loss. However, our results did not reveal any effect of years of experience or background on this accuracy. The effect of health care provider stress, fear of litigation and potentially other confounders in a real life setup may affect their estimation accuracy [6]. Moreover, We have used simulated blood rather than real blood in this research. It is possible that using simulated blood may affect the validity of the research. However, the simulated blood was prepared based on our revision of several research studies where simulated blood was used and after several validating sessions. Finally, this research has proved that education does improve health care providers accuracy in estimating blood loss. We do recommend a follow up study that assesses the effect of the resulting improvement on patients outcome in a real clinical setup. Moreover, validation of this research results in a clinical setting on real patients and blood is needed.

## Conclusion

A simple and consistent education programme can help to overcome typical blood loss estimation inaccuracies. A more sophisticated education programme that includes the use of patients’ clinical conditions could also lead to better estimation results. We recommend the use of a simple, practical education programme that teaches all health care providers to accurately estimate blood loss in labour room setups. More advanced lessons, including the use of patient clinical data in the decision to alert and take action on bleeding patients can be taught in well-equipped and high-quality staff situations.

## Competing interests

The authors declare that they have no competing interest.

## Authors’ contributions

HK, designed the research, wrote the proposal, monitored the research conduction and wrote the final manuscript. HD, MA, ES and NT have contributed to the research conduction, teaching sessions, education scenarios, reviewed and approved the final manuscript. YM and DB have managed the blood loss stations, contributed to participants teaching, reviewed and approved the final manuscript. HT, reviewed the research proposal, performed the statistical analysis, wrote the results, read and approved the final manuscript.

## Pre-publication history

The pre-publication history for this paper can be accessed here:

http://www.biomedcentral.com/1471-2393/14/110/prepub
